# Role of detached podocytes in differentiating between minimal change disease and early focal segmental glomerulosclerosis, can we rely on routine light microscopy?

**DOI:** 10.1007/s40620-022-01456-0

**Published:** 2022-11-09

**Authors:** Mahmoud M. Sobh, Ghada El Kannishy, Fatma Moustafa, Riham Eid, Nashwa Hamdy, Samar Tharwat

**Affiliations:** 1 Mansoura Nephrology and Dialysis Unit (MNDU), Faculty of Medicine, Mansoura University, Mansoura, Egypt; 2 Department of Pathology, Faculty of Medicine, Mansoura University, Mansoura, Egypt; 3 Nephrology Unit, Mansoura University Children’s Hospital, Faculty of Medicine, Mansoura University, Mansoura, Egypt; 4 Rheumatology and Immunology Unit, Internal Medicine Department, Faculty of Medicine, Mansoura University, Mansoura, Egypt

**Keywords:** Minimal change disease, Focal segmental glomerulosclerosis, Nephrotic syndrome, Podocytopathy

## Abstract

**Background:**

Detachment of podocytes represents a turning point in the development of glomerular sclerosis and consequently, of CKD progression. Furthermore, detachment may differentiate minimal change disease (MCD) cases—which have only podocyte effacement—from early focal segmental glomerulosclerosis (FSGS) in which effacement and detachment are observed by electron microscopy. Noteworthy, it is not uncommon for early FSGS to present with clinical presentation and light microscopy (LM) pictures identical to MCD. In our routine practice, we often find cells that lie freely in Bowman’s space by LM. In this study, we try to determine whether these cells are detached podocytes that are worth reporting or just an artifact that can be ignored.

**Methods:**

To the best of our knowledge, no study has discussed the accuracy of LM in detecting detached podocytes by the routinely used stains. We retrospectively selected 118 cases that were diagnosed as MCD by LM, and searched for detached cells in Bowman’s space in their archived, routinely stained LM slides. After that, we tried to find any correlation between the clinical course, detached cells in LM picture and the EM reports.

**Results:**

LM can significantly detect detached podocytes with a positive predictive value of 93%, specificity of 85%, and sensitivity of 46%. Significant correlations were found between detached cells and degree of proteinuria and late steroid resistance.

**Conclusion:**

Detecting detached podocytes by LM is a specific finding that must be reported whenever detected, as it predicts response to steroids and may be able to differentiate MCD from early FSGS by identifying patients who could have podocytopenia.

**Supplementary Information:**

The online version contains supplementary material available at 10.1007/s40620-022-01456-0.

## Introduction

Podocytes are octopus-like cells that constitute one of the cornerstones of the glomerular filtration barrier. Actin cytoskeleton and variable anchoring proteins enable the podocyte to accomplish a number of vital functions, and particularly to prevent proteinuria [1]. Podocytopathy results from different causes that disrupt podocyte function [2]. Regardless of the cause of podocytopathy, lost podocytes are irreplaceable as they are post-mitotic cells. Entering the cell cycle can be catastrophic for the remaining podocytes [3]. This may explain the different prognosis in the two main primary podocytopathies, minimal change disease (MCD) and focal segmental glomerulosclerosis (FSGS). Although both share a great deal of their etiology, patients with FSGS have a worse prognosis as they have detached podocytes, while in MCD, there is only foot process effacement without detached podocytes [4, 5]. Lupus nephritis, membranous nephropathy, IgA nephropathy, diabetic kidney disease, and other glomerular diseases also have loss of podocytes in their pathogenesis and progression [2]. Accordingly, detachment of podocytes has to be routinely evaluated in nephropathology reports. Nonetheless, it is still under-reported even in standardized nephropathology reporting systems [6, 7].

Different approaches have been suggested to document the presence of detached podocytes. Most of them are suitable only for the research setting. A number of factors, including tedious work, inaccessible technologies, special stains, sophisticated software, complex microscopic equipment, or inaccurate results, all hinder their use in routine renal biopsies [8]. Currently, the most widely used tool to investigate podocytes is the transmission electron microscope (EM) [6]. Its results are consistent with intravital multiphoton microscopy [9]. On the other hand, standardized light microscopy (LM) nephropathology reports lacked any comments regarding detached podocytes [6, 7]. Although LM was previously used to count the normally-situated podocytes [10, 11], no one has evaluated its accuracy in detecting detached podocytes by the routine stains. During the examination of LM renal biopsies of patients with MCD, our group has frequently found podocyte-like cells that lie free in the Bowman’s space. Most of those patients were steroid-resistant which is not consistent with MCD. FSGS can present early with a LM picture that is similar to MCD, but with a worse prognosis and poor response to steroids [12]. Could these cells represent detached podocytes? Or is it just an artifact? Can this finding predict the clinical outcome and subsequently affect the treatment decisions? In this article, we will try to provide an evidence-based answer to these questions.

## Materials and methods

We conducted this observational study with a retrospective blinded comparison to a gold standard in Mansoura Nephrology and Dialysis unit (MNDU), during the period from March 2020 to January 2021. We reviewed the files of all patients who underwent renal biopsies in Mansoura university hospitals from 2015 to 2020.

### Inclusion and exclusion criteria

Patients who had proteinuria > 1000 mg/day and LM picture compatible with MCD were included. Patients were excluded if the clinical and LM picture is better explained by secondary glomerulonephritis (GN), such as in the presence of positive ANA, anti-ds DNA, monoclonal gammopathy, HCV, HBV, or DKD. Subjects with LM findings not compatible with MCD and suggestive of alternative diagnoses, including evident FSGS, membranous nephropathy, or crescentic GN, were also excluded.

### Outcomes

Primary goals:

1. To assess the accuracy of LM in detecting detached podocytes by correlation to the EM findings as a gold standard.
2. Correlation between LM detached podocytes and the final diagnosis by EM; i.e., MCD or early FSGS.

Secondary goals:

1. Correlation between LM detached podocytes and clinical characteristics.
2. Descriptive analysis of other relevant pathological and clinical findings.

### Methods

**Fig. 1:**
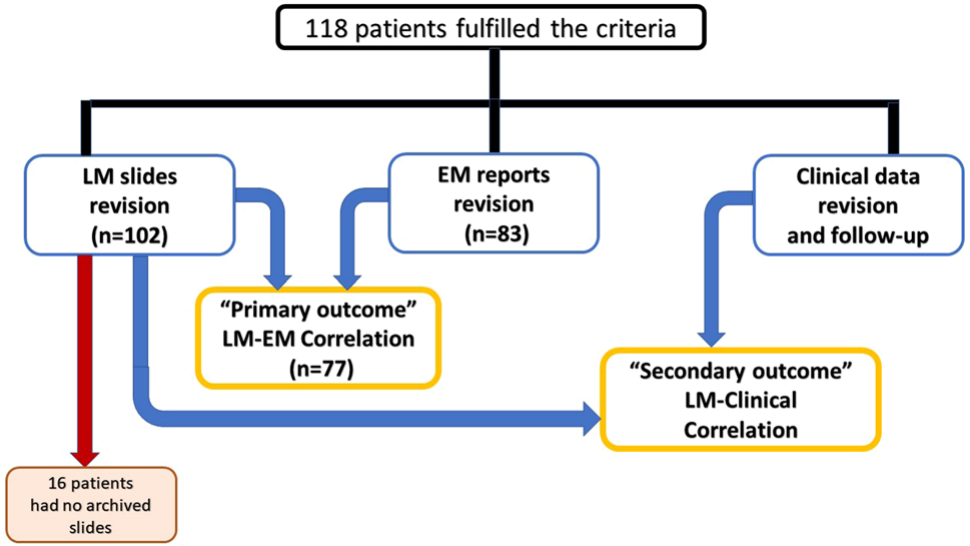
Study flowchart

This study included 118 patients, 102 of whom had available LM slides for re-examination by our expert nephropathologist. To confirm a blinded examination, our nephropathologists had no access to the EM reports. The results of LM re-examination were then correlated to the EM reports, if available. We also revised and collected their clinical data without previously knowing the results of LM or the EM state of podocyte detachment in order to maintain a blinded analysis. Sixteen patients had no available archived LM slides for revision and were excluded from further comparison as shown in Fig. 1, but they were included in the baseline characteristics descriptions.

#### LM revision

**Fig. 2:**
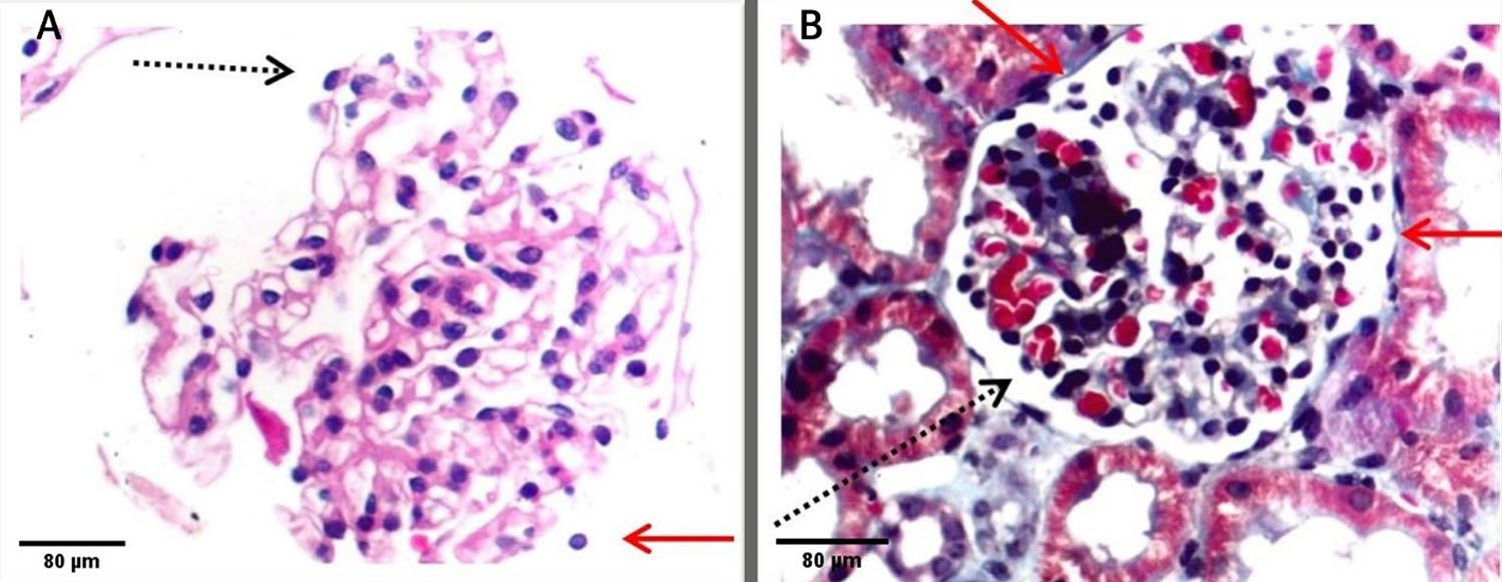
Examples of detached cells. The dotted arrows show normal attachment of the podocyte, while the red arrows show a detached podocyte

LM revision involved searching for the presence or absence of detached cells that have similar characteristics to podocytes, with rounded to globular-shaped rather than flat to spindle-shaped cells, found freely in the urinary space. Figure 2 shows an example of this finding. The presence of this finding was then graded as shown in Table 3.

#### EM comparison

We compared the LM findings to the EM results by searching for detached podocytes. We also reviewed other abnormalities that were revealed by EM such as the final diagnosis, podocyte effacement and its degree, and basement membrane (BM) abnormalities as basketweave appearance, thinning, or thickening according to reference levels in adults and children [13, 14].

#### Demographic, clinical, laboratory and follow-up data

Demographic data were retrieved, including age and sex. Adults were defined as age 18 years or older. Clinical and laboratory data were also reported including associated comorbidities, family history of nephrotic syndrome (NS), blood pressure, serum creatinine, serum albumin, hematuria, and proteinuria, Patients were classified according to KDIGO Clinical Practice Guidelines for GN [15]. Early response to steroids was assessed after 4 months, and to cyclosporin (CSA) after 6 months of treatment. Late response to both was assessed after 1 year of treatment.

#### Statistical analysis

Data were analyzed using the Statistical Package of Social Sciences (SPSS) version 25 for windows (SPSS, Inc., Chicago, IL, USA). The distribution of tested variables was examined by the Kolmogorov–Smirnov test for normality. Data were presented using mean and standard deviation (SD) for parametric variables or median and interquartile range (IQR) for non-parametric variables, and numbers and percentages for qualitative variables. The significance of differences between continuous variables was determined by the independent samples *t*-test for variables with normal distribution and Mann–Whitney test for not normally distributed variables, as appropriate. Chi-square or Fisher’s exact test was used for comparison between qualitative variables, as appropriate. One-way analysis of variance (ANOVA) was used to examine mean differences between more than two normally distributed variables. Pearson’s correlation or Spearman’s correlation was used to examine the correlation between continuous variables. Cohen’s Kappa analysis was carried out to assess the agreement between LM and EM. Sensitivity, specificity, accuracy, positive and negative predictive values were calculated by cross-tabulation. Multivariate logistic regression analysis was performed to determine the ability of LM-identified detachment to predict late steroid resistance. Besides LM-identified detachment, the model also included serum albumin, age, and sex. A *p* value ≤ 0.05 was considered significant.

## Results

### Baseline characteristics

This study included 118 patients who fulfilled our criteria. Combined archived LM slides and EM reports were available for 77 patients, as shown in Fig. 1. Documented clinical follow-up duration ranged from 6 months to 17 years, with a median of 3 years and IQR was 1–5 years. A family history of nephrotic syndrome was present only in seven patients. Eight patients had sCr above 1.5 mg/dl at presentation. Full nephrotic range proteinuria occurred at presentation in 100 patients, as shown in Table 1. Most of the patients were steroid-resistant and CSA-sensitive with different degrees of response as illustrated in Table 2. EM results dramatically changed the diagnosis from MCD by LM to multiple other diagnoses; features of early FSGS—particularly detachment—were found in about 75% of cases. Of note, basement membrane abnormalities indicating Alport syndrome were observed in nearly 25% of EM reports which decreased the prevalence of steroid responders in our cohort. All of them showed steroid resistance except two partial responders and two frequent relapsers. Light and electron microscopy data are shown in Table 3.

**Table 1:** Baseline demographic, clinical and laboratory data (*n* = 118)

Variable	Total (*n* = 118)	Adult (*n* = 50)	Children (*n* = 68)
Age at onset, years	9 (4–27)	30 (17–42)	6 (3–10)
*Sex*			
Males	68 (58%)	29 (58%)	39 (57%)
Females	50 (42%)	21 (42%)	29 (43%)
*Associated comorbidities*			
Diabetes mellitus	3 (2.5%)	2 (4%)	1 (1.5%)
Hypertension	4 (3%)	2 (4%)	2 (3%)
Diabetes and hypertension	3 (2.5%)	3 (6%)	0 (0%)
Thyroid disorders	2 (1.7%)	2 (4%)	0 (0%)
Hematologic malignancy	1 (1%)	1 (2%)	0 (0%)
Chronic liver disease	2 (1.7%)	1 (2%)	1 (1.5%)
Family history of NS	7 (6%)	3 (6%)	4 (6%)
Hypertension at presentation	14 (12%)	10 (20%)	4 (6%)
Follow up duration (years)	3 (1.2–5)	5 (1–12.5)	3 (2–4)
*S. cr (mg/dl):*	0.6 (0.5–0.8)	0.8 (0.6–1.1)	0.5 (0.5–0.7)
Patients with s. cr ≤ 1 mg/dl	102 (86%)	39 (78%)	63 (93%)
Patients with s. cr 1.1—1.5 mg/dl	8 (7%)	5 (10%)	3 (4%)
Patients with s. cr > 1.5 mg/dl	8 (7%)	6 (12%)	2 (3%)
S. albumin (gm/dl)	2 (1.8–2.7)	2.5 (1.9–2.9)	2 (1.8–2.3)
*Proteinuria*			
24-h urine protein (gm/day)	2.5 (2–4)	5 (2.5–7)	2 (2–3)
Subnephrotic proteinuria	18 (16%)	10 (20%)	8 (12%)
Nephrotic proteinuria	100 (84%)	40 (80%)	60 (88%)
*Hematuria*			
Microscopic hematuria (occasional)	7 (6%)	1 (2%)	6 (9%)
Microscopic hematuria (persistent)	1 (1%)	0 (0%)	1 (1%)
Macroscopic hematuria	2 (2%)	0 (0%)	2 (3%)

Quantitative variables are expressed as median (interquartile range) for non-normally distributed data. Qualitative variables are expressed as number (percentage)

*BP* blood pressure, *HTN* hypertension, *NS* nephrotic syndrome, *S. Cr* serum creatinine, *S. Alb* serum albumin

**Table 2:** Response to steroids and cyclosporine in the studied cases

Response to steroids	Total (*n* = 89)	Adult (*n* = 30)	Children (*n* = 59)
*Early steroid response*			
Complete remission	21 (24%)	5 (17%)	16 (27%)
Partial remission	9 (10%)	8 (27%)	1 (2%)
Steroid dependent	10 (11%)	2 (7%)	8 (14%)
No remission	49 (55%)	15 (50%)	34 (58%)
*Late steroid response*			
Complete remission	1 (1%)	0 (0%)	1 (2%)
Partial remission	6 (7%)	6 (20%)	0 (0%)
No remission	61 (69%)	17 (57%)	44 (75%)
Steroid dependent	11 (12%)	3 (10%)	8 (14%)
Infrequent relapsers	1 (1%)	1 (3%)	0 (0%)
Frequent relapsers	9 (10%)	3 (10%)	6 (10%)

Response to CSA	Total (*n* = 55)	Adult (*n* = 26)	Children (*n* = 29)
*Early CSA response*			
Complete remission	32 (58%)	17 (65%)	14 (48%)
Partial remission	21 (38%)	7 (27%)	15 (52%)
CSA dependent	0 (0%)	0 (0%)	0 (0%)
No remission	2 (4%)	2 (8%)	0 (0%)
*Late CSA response*			
Complete remission	23 (42%)	12 (46%)	11 (38%)
Partial remission	21 (38%)	7 (27%)	14 (49%)
No remission	3 (5.5%)	2 (8%)	1 (3%)
Infrequent relapsers	4 (7%)	2 (8%)	2 (7%)
Frequent relapsers	3 (5.5%)	2 (8%)	1 (3%)
CSA dependent	1 (2%)	1 (4%)	0 (0%)

Qualitative variables are expressed as numbers (percentage)

*CSA* Cyclosporine A

**Table 3:** Light and electron microscopy data in the revised slides (*n* = 102)

LM findings	Total (*n* = 102)	Adult (*n* = 41)	Children (*n* = 61)
Podocyte detachment	44 (43%)	17 (41%)	27 (44%)
*Percentage of biopsied glomeruli that have detached podocytes*			
Grade 1 (Occasional): < 25%	39 (89%)	14 (82%)	25 (93%)
Grade 2 (Focal): 25% to 50%	4 (9%)	2 (12%)	2 (7%)
Grade 3 (Diffuse): > 50%	1 (2%)	1 (6%)	0 (0%)
*Mean detached cells / affected glomeruli *^*x*^			
1 cell/glomerulus	28 (64%)	12 (71%)	16 (59%)
2 cells/glomerulus	10 (23%)	5 (29%)	5 (19%)
3 cells/glomerulus	3 (7%)	0 (0%)	3 (11%)
4 cells/glomerulus	2 (4%)	0 (0%)	2 (7%)
5 cells/glomerulus	1 (2%)	0 (0%)	1 (4%)

EM findings	Total (*n* = 83)	Adult (*n* = 34)	Children (*n* = 49)
Podocyte detachment	69 (83%)	7 (21%)	42 (86%)
*Foot process effacement*	69 (83%)	28 (82%)	41 (84%)
Partial effacement	24 (35%)	5 (18%)	19 (46%)
Diffuse effacement	45 (65%)	23 (82%)	22 (54%)
*Final diagnosis by EM*			
Early FSGS	48 (58%)	16 (47%)	32 (65%)
MCD	9 (11%)	4 (12%)	5 (10%)
Alport	4 (5%)	1 (3%)	3 (6%)
Alport + Early FSGS	16 (19%)	8 (23%)	8 (16%)
Alport + MCD	1 (1%)	0 (0%)	1 (2%)
Membranous GN	3 (4%)	3 (9%)	0 (0%)
Thin BM disease	1 (1%)	1 (3%)	0 (0%)
DN + detached podocytes	1 (1%)	1 (3%)	0 (0%)

Qualitative variables are expressed as number (percentage)

*MCD* minimal change disease, *FSGS* focal segmental glomerulosclerosis, *EM* electron microscopy, *LM* light microscopy, *BM* basement membrane

*x*: Calculated by the following equation: $\frac{\text{Sum of detached cells for all glomeruli}}{\text{Number of affected glomeruli}}$

### LM-EM correlation

EM reports were reviewed and correlated with LM slides in 77 patients, as the remaining 6 patients had no archived LM slides. LM and EM were in agreement on 29 patients as having detached podocytes and on 12 patients as having no detached podocytes. However, LM found detached podocytes in two more patients who were identified as having no detached podocytes by EM. LM also missed 34 patients who were identified as having detached podocytes by EM. LM performance parameter in detecting detached podocytes in comparison to EM was as follows; Kappa value was 0.168. LM had a positive predictive value of 93% and specificity of 85%. On the other hand, sensitivity was 46%, negative predictive value was 26% and accuracy was 53% (*X*^2^ = 4.8, *p* value = 0.028). Table 4 illustrates further LM/EM/clinical correlations. Seven cases had higher degrees of detachment; EM confirmed podocyte detachment in all of them. Regarding the final diagnosis by EM, 10 cases had a picture of MCD by EM, none of them had detached cells by LM as shown in Table 4.

**Table 4:** Correlation of primary and secondary outcomes with microscopic findings

***Primary outcome “LM/EM correlation”***				
*1. Agreement of LM detachment and EM detachment*^a^	Patients with EM detachment	Patients with no EM detachment	Total	
Patients with LM detachment	29	2	31	
Patients with no LM detachment	34	12	46	
Total	63	14	77	
*2. Agreement of LM with the final EM diagnosis*^b^	MCD by EM	Early FSGS by EM	Others	
Patients with LM detachment	0	29	2	
Patients with no LM detachment	10	30	6	
Total	10	59	8	
***Secondary outcome “LM/clinical correlation”***				
*1. Univariate Correlation with degree of proteinuria*^c^	Patients with LM detachment	Patients with no LM detachment	X^2^	*p* value
Sub-nephrotic	3 (7%)	14 (24%)	5.4	0.02
Nephrotic	41 (93%)	44 (76%)		
*2. Multivariate logistic regression analysis for prediction of late steroid resistance*^d^				
Predictor	Odds ratio (confidence interval)	*p* value		
Presence of LM detachment	4.461 (1.170–17.006)	0.028		

*EM* electron microscopy, *LM* light microscopy, *PPV* positive predictive value, *NPV* negative predictive value

*By Fisher’s Exact Test, MCD: minimal change disease, FSGS: focal segmental glomerulosclerosis a: *p* value is 0.028, b: *p* value is 0.004, c: other variables are illustrated in supplementary tables, d: This model also included age, sex, serum albumin besides LM detachment. The latest was the only statistically significant variable in this model was the presence of detachment in the light microscopic picture

### LM clinical correlation

Detached cells observed by LM had a significant positive correlation with the degree of proteinuria at presentation and a significant positive correlation with HTN in the children’s group together with a negative correlation with serum albumin in the adult group. Sub-classifications of results for adults and children are shown in the supplementary materials (Tables 1S–9S). Multivariate logistic regression was performed to ascertain the effects of detachment by LM, serum albumin at onset, age, and sex on the likelihood that participants would exhibit late steroid resistance. Of the four predictor variables, only detachment observed by LM was a statistically significant independent predictor of steroid resistance. Participants with LM detachment had 4.5 times higher odds of exhibiting steroid resistance, as shown in Table 4. Our archive was reviewed again for the last 2 years (2021–2022) to see whether any members of the studied cohort had a follow-up biopsy. Only one patient underwent a repeat biopsy due to rising serum creatinine, perisitant proteinuria and lack of response to immune-suppressive drugs. The first biopsy showed no abnormalities apart from effacement and detached podocytes in both light and electron microscopy pictures, which we hypothesized to be early FSGS. The second biopsy showed a manifest FSGS picture in the form of 18 glomeruli (10 globally and 3 segmentally sclerotic) along with detached podocytes by LM.

**Fig. 3:**
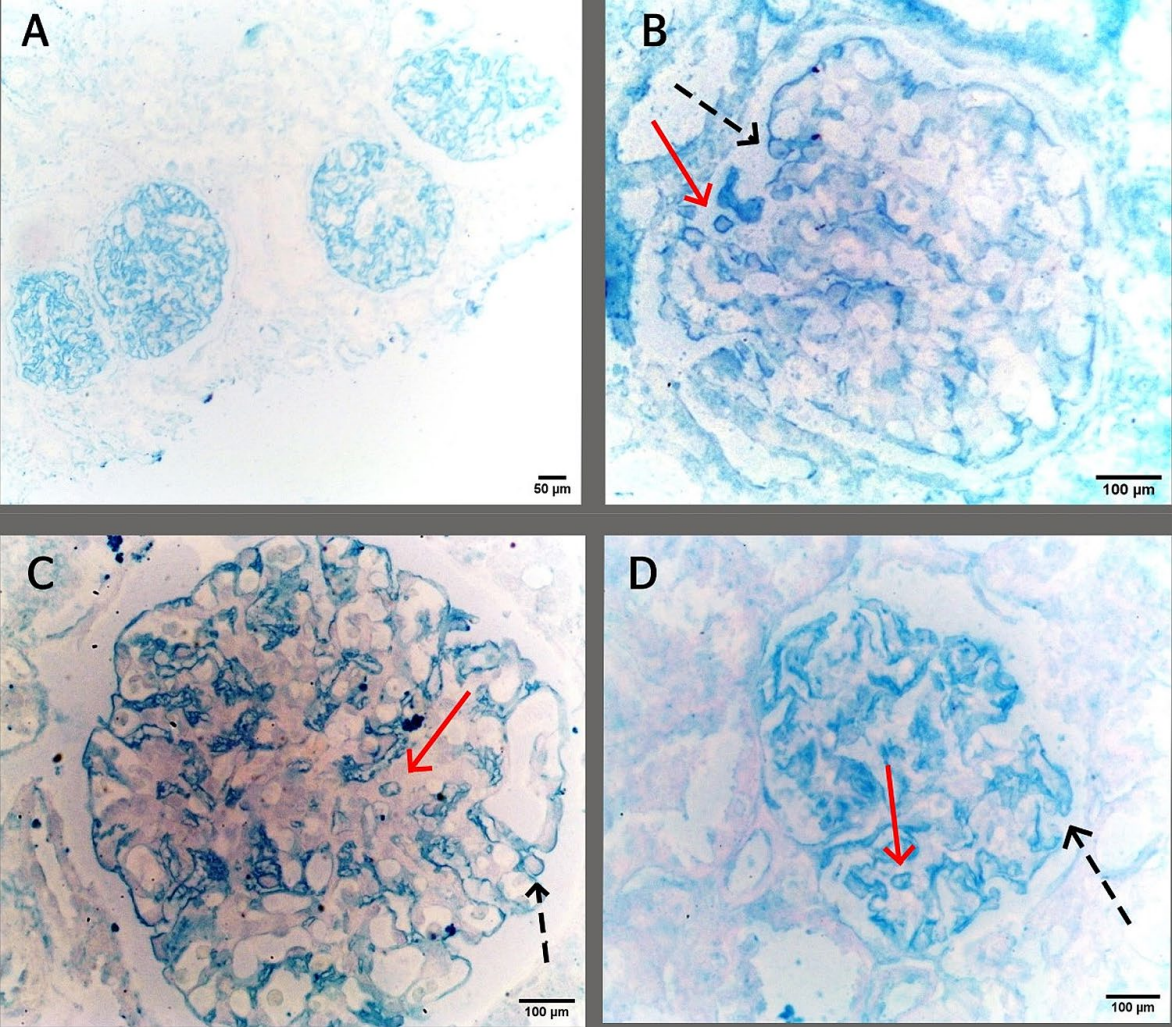
Detached cells detected by colloidal-iron stain. **A** 4 glomeruli stained by colloidal iron delineating the podocalyxin coat of podocytes, **B**–**D** red arrows refer to detached podocytes surrounded by clear halo, and black dashed arrows refer to normally attached podocytes

We used a special podocyte stain to clearly identify the nature of the detached cells. We had 29 patients with confirmed detachment by routine light and electron microscopy. Their paraffin blocks were searched for any residual glomerular tissue to be stained by a special podocalyxin stain (colloidal iron) [16–18]. Of the nine patients who were eligible for this revision, the special stain confirmed detached cells in five of them, as shown in Fig. 3.

## Discussion

On repeat biopsy, it is not uncommon to find cases of early FSGS in patients who were initially diagnosed with MCD [19]. Thus, this is one of the confusing clinical scenarios in nephrotic syndrome, when LM reveals no abnormalities in the glomerulus and the patient’s response to steroid therapy is weak. EM is not always available either due to higher costs or to the longer time needed to carry out the examination and to get the results. Accordingly, any significant finding in the LM picture should never be neglected, as it may help predict the prognosis and explain the disease pathogenesis, ultimately affecting the treatment decisions. Moreover, with the recent discoveries on the role of podocytes in the pathogenesis of this group of glomerulonephritis, it has become essential to fully describe podocyte abnormalities in both LM and EM reports. One of the limitations of the available podocytopathy studies is the limited number of patients who had EM examinations [20, 21]. If we could assess their podocytes by LM, this might open the door for clinical studies with a larger sample size. In our examination of kidney biopsies of patients with NS without abnormalities in the LM picture, we observed few cells in the urinary space without attachment to the BM, as shown in Fig. 2. We hypothesized that these cells were detached podocytes and that this could direct the diagnosis toward early FSGS instead of MCD. This differentiation works in harmony with the Barisoni classification of primary podocytopathy which considers lost podocytes as the differentiating factor between FSGS and MCD [4].

Thus, we aimed to assess the role of LM in detecting detached podocytes. To the best of our knowledge, no study has discussed the ability of LM to detect podocyte detachment by routinely used stains. All the available studies discussed podocyte detachment as described with  EM or by special stains or by counting all the currently available podocytes within the glomerulus [9, 10, 16, 22]. We made the hypothesis that if we succeeded in confirming the ability of LM to detect detached podocytes, this would add a new, inexpensive, and easy method for in-depth evaluation of LM sections in patients with apparent MCD.

Several previous studies that tackled MCD and FSGS had a major risk of bias, which is related to the absence of EM examination. Accordingly, a large percentage of the included patients might have been misdiagnosed as MCD [20, 21, 23, 24]. EM is essential for unmasking cases of early FSGS that may present as MCD. MCD is not well studied in Egyptian adults. We retrieved all cases with the previously mentioned dilemma “apparent MCD by LM” in the period from 2015 to 2020. There were 118 patients; 50 adults and 68 children. In our study, of the 83 EM biopsies, 74 (89%) showed findings other than MCD, 69 (83%) showed podocyte detachment, 48 (57.8) were diagnosed as early focal segmental glomerulosclerosis that was not revealed until EM was done, only 9 (10.8%) were diagnosed as MCD, 25 (28%) had BM abnormalities, 3 (3.6%) had an early membranous pattern of GN that was not revealed until EM was done and 22 (24%) had EM characteristics of Alport syndrome.

To assess the accuracy of LM in detecting these detached cells, we had to compare the results to a gold standard. Thus, we compared the LM findings to EM and we also correlated them to the response to steroid treatment, as steroid response is considered a better predictor of early FSGS than EM [25].

In our study, the presence of detached cells in the LM picture was significantly correlated with the degree of proteinuria; cases with detached podocytes had a higher incidence of nephrotic range proteinuria. This agrees with the results obtained by Hanamura and colleagues who reported that detached podocytes detected in urine samples were associated with higher proteinuria [10]. Hanamura and colleagues also failed to find any association between urinary detached podocytes and BP, hematuria, or eGFR; these findings were similar to our study.

Response to steroid treatment is expected to be better in cases without podocyte detachment, as they encompass cases of MCD. It is reported that response to steroids is a valuable tool to differentiate between MCD and FSGS [25]. In the present study, multivariate logistic regression showed a similar finding; only podocyte detachment by LM was a statistically significant independent predictor of steroid resistance. Cases with LM detachment had 4.5 times higher odds to exhibit steroid resistance. Taneda et al. and Abumregha et al. also reported a higher incidence of steroid resistance among FSGS cases in comparison to MCD [26, 27].

Detached podocytes in LM were confirmed by EM in 93% of cases. When there was detachment in ≥ 25% of biopsied glomeruli or mean detached cells per affected glomeruli > 1, EM confirmed podocyte detachment in 100% of the cases. This indicates a high specificity and high positive predictive value of LM, which is expected, since LM was used to count the total number of podocytes per glomerulus, and podocytes were identified as cells that reside within the tuft of the glomerulus but outside the glomerular basement membrane [10, 11]. Podocyte detachment by LM was not confirmed in two cases by EM. This can be explained by the focal distribution of the lesions. Routine EM preparations choose only one or two glomeruli for examination; if the examined glomerulus was normal, the results could be non-representative, while LM is more representative because it has a larger sample size. Although the positive predictive value was good, there were several false-negative cases, i.e., 34 out of 63 cases with no LM detachment, and this led to low sensitivity (46%). Thus, Cohen’s Kappa analysis showed slight agreement between LM and EM in assessing detached podocytes, even with a significant *p* value of 0.028.

This study focuses on determining the diagnostic value of detecting those detached cells which are assumed to be podocytes detaching from the basement membrane. To be able to differentiate early FSGS from MCD based on this LM finding, there are two steps for verification. The first step is to ensure that these cells actually are podocytes (which has been done successfully in this study) by reviewing the EM and searching for detached podocytes. Standardized reporting of these cells is mandatory as they have a high positive predictive value for the EM picture of detached podocytes, and this was the main aim of our study. Such finding is essential not only in the pathology of FSGS but also in multiple other glomerular disorders including diabetic nephropathy, membranous nephropathy, lupus nephritis, and others.

The second step should be to ensure that patients with detached cells have FSGS and not MCD, and this is the debatable point. Detachment is the primary pathogenic step that leads to the formation and progression of sclerosis. Animal models of podocyte injury using diphtheria toxins or Puromycin confirm this pathogenetic pathway; animals with no, or minimal podocyte detachment, resemble MCD cases. Detachment—if present—should be below the compensation limit of the glomerulus. Increasing the dose and duration of the toxic substance will increase the podocyte injury beyond the compensation limits leading to FSGS [28]. In humans, Royal et al., classified renal biopsies from > 200 patients with minimal change disease or FSGS according to ultrastructural pathological changes into six clusters. Clusters 4, 5, and 6, which included the majority of FSGS cases, had the highest probability of including detached podocytes and vice versa [29]. Additionally, in a recent study, Zee et al. described the detachment of podocytes in LM images as a halo around the podocytes. This halo was present in 19% of FSGS cases and absent in MCD and MCD-like cases, with clinical correlations similar to those found in our study [30]. Furthermore, Ahn and Bomback stated that in their approach to diagnosing primary podocytopathy: “There are hallmark differences in the histologic appearances of MCD and FSGS, which in turn represent distinct pathogenic models after initial podocyte injury (e.g., no change in podocyte number in MCD vs podocyte detachment and death in FSGS)” [5]. A similar concept was mentioned in Wiggins’ spectrum of podocytopathy: “Podocytes are not lost into the urine in MCD with the maintenance of the normal podocytes numbers compatible with the excellent long-term prognosis for renal function”.

To confirm the diagnostic and prognostic value of podocyte detachment we need a study that has follow-up biopsies for patients who were initially diagnosed as MCD, after which the second biopsy showed FSGS. Thus the first biopsies have to be reviewed for the presence or absence of detached cells. In our cohort, we found only one patient with repeat biopsy; the first biopsy showed MCD with detached cells, and his second biopsy showed FSGS. Of course, one patient is not enough. Thus, we can not be sure that if detached cells are detected, the diagnosis is FSGS. A helpful approach for such patients is to confirm the state of podocytopenia by a quantitative assessment of the remaining podocytes using special podocyte stains that were not feasible in our study. Even though we cannot confirm the diagnosis of FSGS, these cases would be better classified as podocytopathy with detached podocytes rather than MCD, since podocyte loss is a change that should not be reported as “Minimal”.

One of the unexpected findings in the present study is the high incidence of Alport syndrome features in EM results. The majority of similar studies reported a lower incidence than in our study where approximately 25% of cases showed features of Alport syndrome [20, 24, 31]. This unusual finding may be explained by the presence of a different genetic background in our cohort with different inherited mutations and consanguinity patterns, in addition to differnce in inclusion and exclusion criteria between studies, and selective availability of EM. In our community, previous studies reported Alport syndrome to be the most common cause of hematuria in Egyptian children and Alport syndrome represents > 16% of the original kidney disease in transplanted children [13, 32]. Alport syndrome mostly presents with hematuria, but it may present with nephrotic syndrome even in the absence of hematuria [33, 34].

There is a grey area between cases of FSGS and cases with a nephrotic presentation of Alport syndrome. Effacement of podocyte foot processes and podocytopenia were reported in cases of Alport syndrome [35, 36], but Tsuji et al. reported that no podocyte detachment was present [37]. Thus, these detached cells may indicate the concomitant presence of FSGS, a podocytopathy that is characterized by podocyte detachment and podocytopenia, according to the taxonomy of Barisoni and colleagues [4]. The presence of detachment in Alport syndrome may further increase the interest of the studied approach. Based on this fact, noticing a detached podocyte can distinguish MCD from early Alport syndrome, that might present with no abnormality by LM, in the absence of other differentiating parameters.

To the best of our knowledge, this is the first study that tries to validate the ability of LM to detect detached podocytes by routine stains. Moreover, this study discusses a common finding in nephropathology practice and tries to find an evidence-based answer to the following question: “should the nephropathologist ignore this detached cell or does it deserve to be reported?” Particularly, he/she does not have to use expensive special stains or sophisticated techniques to report such essential findings when it is present in the routine LM examination. Additionally, MCD has not been adequately studied among the Egyptian adult population. This is the first study to combine clinical, laboratory, and pathological (LM and EM) characterization in adult MCD patients in Egypt using EM in the majority of the studied population. Most studies that evaluated FSGS cases ignored the cases that were diagnosed as apparently MCD by LM. Our study is one of the few that focused on the hidden spectrum of FSGS cases and described its characteristic presentation, laboratory, and pathological features, and response to treatment.

Our study has limitations. First, it was retrospective. If we had the chance to carry out a prospective study, we would be able to add a few items to the studied parameters such as correlation with urinary podocytes. Second, limited resources led to the absence of genetic analysis for the studied population. Discovering the genetic background could help to better classify and understand the disease pathogenesis. Particularly, genetic podocytopathy does not always present with a positive family history or syndromic features, it has even improved with the administration of immunosuppressive agents in a few occasions [33, 38]. A higher number of MCD patients, particularly those who are steroid-responsive, would add more statistical power, but in routine practice, those cases usually do not proceed to kidney biopsy. This explains why we have a higher prevalence of steroid resistance among children in this study, as well as a high incidence of Alport syndrome in our series. Further in-depth studies of cases of Alport syndrome would solidify the diagnosis, not only by genetic analysis but also through audiograms, lens examination, special collagen stains for basement membrane, and examination of family members, all of which might help achieve a better understanding of such an under-reported disease. Podocytopenia may result from the actual loss of podocytes by detachment or from increased glomerular size with a fixed number of podocytes which is known as relative podocytopenia. Our study tackled only the first mechanism. Special podocyte stains and stereological methods for quantifying podocytopenia can indeed be more accurate in assessing and quantifying podocyte loss, but EM and response to steroids were the best available gold standards for comparison with our finding of LM detachment. Moreover, histological approaches might miss some cases due to sampling error. Response to steroids was reported by KDIGO GN 2021 guidelines as a better differentiating tool in such conditions. Moreover, there were concerns regarding the proper identification of podocytes by routine LM stains, as these free cells might be detached tubular, inflammatory, or even parietal epithelial cells. Morphological characteristics of podocytes are distinct from parietal cells, which are flat and not rounded, and from tubular cells that should normally be shed distally with the flow of the filtered urine away from the glomerulus. Moreover, the pathogenesis of this group of diseases includes only detachment of podocytes and not parietal cells without inflammatory cellular infiltration in the Bowman’s space.

We suggest that future studies should prospectively include staining for one or two special podocyte markers (such as ZO-1, WT-1, synaptopodin, CD2AP, podocin, podocalyxin, nephrin) to ensure better identification of these detached cells. The same approach can be followed in cases where LM detects detached cells and EM reports no detached podocytes. In the current study, we retrospectively tried to confirm our findings by using a special podocyte stain. Out of 29 patients who had detached cells on routine light and electron microscopy, 9 patients had sufficient glomerular tissue in their paraffin blocks. After staining by colloidal iron, 5 patients had detached cells. Considering the 3-dimensional configuration of the glomerulus, detached cells may be present in one section and not present in another within the same glomerulus. This explains why not all patients were positive by the special stain and adds to the low sensitivity of our finding. On the other hand, detached cells that were stained by colloidal iron in the five patients confirm the relatively high specificity of this test.

In our study, five cases showed binucleated podocytes indicating mitotic catastrophe. EM showed detached podocytes in all of them. Among those five patients, only one had positive LM detachment as well. Future studies may also include an assessment of the viability of podocytes while still anchored to the outer glomerular basement membrane; apoptosis, mitotic catastrophe, necroptosis, pyroptosis, or vacuolizations can be explored by LM among those patients with apparently normal glomerulI.

## Conclusion

LM detection of detached podocytes is a specific finding suggesting the presence of FSGS. The presence of detached podocytes must be reported whenever detected, as it might be able to differentiate MCD from early FSGS, by identifying patients who may have podocytopenia. It can also be used to predict late response to steroids in patients with MCD or early FSGS. 

## Supplementary Information

Below is the link to the electronic supplementary material.

Supplementary file1 (DOCX 35 kb)

## Data Availability

The datasets generated during and/or analyzed during the current study are available from the corresponding author on reasonable request.

## Abbreviations

FSGS: Focal segmental glomerulosclerosis
MCD: Minimal change disease
LM: Light microscopy
EM: Electron microscopy
GN: Glomerulonephritis
IQR: Interquartile range
SD: Standard deviation
CSA: Cyclosporin A
BM: Basement membrane
