# Supplementary material for: Role of detached podocytes in differentiating between minimal change disease and early focal segmental glomerulosclerosis, can we rely on routine light microscopy?
Source: J Nephrol. 2022 Nov 9;35(9):2313–24. doi: 10.1007/s40620-022-01456-0 (PMC9700609; doi:10.1007/s40620-022-01456-0)
Supplement: Supplementary file 1 — Supplementary file1 (DOCX 35 kb) [file 40620_2022_1456_MOESM1_ESM.docx]

# **Supplementary Materials**

## LM-Clinical correlations:

**Table 1S: Correlation between light microscopy findings of detached podocytes and clinical data**

| **Clinical data** | **Podocyte detachment by LM** | | | **χ^2^/FET or**  **Z-value** | | **P-value** |
| --- | --- | --- | --- | --- | --- | --- |
|  | **No** | **Yes** |  | |  | |
| **BP at presentation**  Normal  High | 53 (91)  5 (9) | 37 (84)  7 (16) | 1.2**^χ^** | | 0.25 | |
| **S. Cr at onset** (mg/dl)  ≤ 1 (mg/dl)  1.1 - 1.5 (mg/dl)  > 1.5 (mg/dl) | 0.6 (mg/dl)  (0.5-0.8)  51 (88)  3 (5)  4 (7) | 0.6 (mg/dl)  (0.4-0.8)  37 (84)  4 (9)  3 (7) | -0.55^z^  FET | | 0.58  0.76 | |
| **S. Cr after follow up** (mg/dl)  ≤ 1 (mg/dl)  1.1 - 1.5 (mg/dl)  > 1.5 (mg/dl) | 0.75 (mg/dl)  (0.6-0.9)  21 (88)  1 (4)  2 (8) | 0.7 (mg/dl)  (0.6-1)  17 (77)  0 (0)  5 (23) | -0.25^z^  FET | | 0.8  0.23 | |
| **Hematuria**  Microscopic occasional  Microscopic persistent  Macroscopic | 5 (9)  0 (0)  1 (1) | 2 (5)  1 (2)  1 (2) | FET | | 0.66 | |
| **Proteinuria**  Subnephrotic  Nephrotic | 14 (24)  44 (76) | 3 (7)  41 (93) | 5.4**^χ^** | | **0.02** | |
| **24-hour urinary protein** (gm/dl) | 2.6 (2 - 3.75) | 2 (1.8-3) | -1.36^z^ | | 0.17 | |
| **S. Albumin** (gm/dl) | 2.25 (1.9-2.9) | 2 (1.6-2.5) | -1.78^z^ | | 0.076 | |

Quantitative variables are expressed as median (interquartile range) for non-normally distributed data. Qualitative variables are expressed as number (percentage). BP: blood pressure, HTN: hypertension, S. Cr: serum creatinine, FET: Fisher’s exact test, s. albumin: serum albumin, z: Z-value, χ: chi square value

**Table 2S: Correlation between light microscopy findings of detached podocytes and response to steroid treatment in studied cases**

| **Response to steroids** | **Podocyte detachment by LM** | | **χ^2^**  **or**  **^FET^** | | **P-value** | |
| --- | --- | --- | --- | --- | --- | --- |
|  | **No** | **Yes** | |  | |  |
| **Early steroid response** (4 groups)  Complete remission  Partial remission  Steroid-dependent  No remission | 14 (34)  3 (7)  5 (12)  19 (46) | 5 (15)  3 (9)  5 (15)  20 (60) | | FET | | 0.31 |
| **Early steroid response** (3 groups)  Complete remission  Partial remission + steroid-dependent  No remission | 14 (34)  8 (20)  19 (46) | 5 (22)  8 (24)  20 (60) | | 3.4 | | 0.17 |
| **Early steroid response** (2 groups)  Sensitive  Resistant | 22 (54)  19 (46) | 13 (39)  20 (61) | | 1.5 | | 0.22 |
| **Late steroid response** (6 groups)  Complete remission  Partial remission  No remission  Infrequent relapsers  Frequent relapsers  Steroid-dependent | 1 (2)  2 (5)  25 (61)  1 (2)  6 (15)  6 (15) | 0 (0.0)  2 (6)  26 (79)  0 (0.0)  1 (3)  4 (12) | | FET | | 0.39 |
| **Late steroid response** (3 groups)  Complete remission + infrequent relapsers  Partial remission +frequent relapsers+ St. depend.  No remission | 2 (5)  14 (34)  25 (61) | 0 (0)  7 (21)  26 (79) | | FET | | 0.2 |
| **Late steroid response** (2 groups)  Sensitive  Resistant | 16 (39)  25 (61) | 7 (21)  26 (79) | | 2.7 | | 0.10 |

Qualitative variables are expressed as N; number (percentage). FET: Fisher’s exact test

**Table 3S: Correlation between light microscopy findings of detached podocytes and response to cyclosporine**

| **Response to cyclosporine A** | **Podocyte detachment by LM** | | **χ^2^**  **or**  **^FET^** | **P-value** |
| --- | --- | --- | --- | --- |
|  | **no** | **Yes** |  |  |
| **Initial CSA response** (3 groups)  Complete remission  Partial remission  No remission | 12 (57)  9 (42)  0 (00) | 12 (55)  9 (41)  1 (4.5) | FET | 1 |
| **Late CSA response** (6 groups)  Complete remission  Partial remission  No remission  Infrequent relapsers  Frequent relapsers  CSA dependent | 10 (47)  8 (38)  1 (5)  0 (0)  1 (5)  1 (5) | 9 (41)  10 (45)  1 (4.5)  1 (4.5)  1 (4.5)  0 (11) | FET | 0.94 |
| **Late CSA response** (2 groups)  Sensitive  Resistant | 20 (95)  1 (5) | 21 (95.5)  1 (4.5) | FET | 0.47 |
| **Late CSA response (3 groups)**  Complete remission + infrequent relapsers  Partial remission+ frequent relapsers+ CSA depend.  No remission | 10 (48)  10 (48)  1 (4) | 10 (45)  11 (50)  1 (5) | FET | 1 |

Qualitative variables are expressed as N; number (percentage). CSA: cyclosporine A, FET: Fisher’s exact test

**Table 4S: Correlation between light microscopy findings of detached podocytes and clinical data in adults**

| **Clinical data** | **Podocyte detachment by LM** | | **χ^2^/FET or**  **Z-value** | **P-value** |
| --- | --- | --- | --- | --- |
|  | **no** | **Yes** |  |  |
| **BP at presentation**  Normal  High | 19 (79)  5 (21) | 14 (82)  3 (18) | FET | 1 |
| **S. Cr at onset** (mg/dl)  ≤ 1 (mg/dl)  1.1 - 1.5 (mg/dl)  > 1.5 (mg/dl) | 0.8 (mg/dl) (0.6-0.98)  19 (79)  2 (8)  3 (13) | 0.8 (mg/dl) (0.6-1.2)  13 (77)  2 (12)  2 (12) | - 0.4 ^z^  FET | 0.69  1 |
| **S. Cr after follow up** (mg/dl)  ≤ 1 (mg/dl)  1.1 - 1.5 (mg/dl)  > 1.5 (mg/dl) | 0.9 (mg/dl) (0.77-0.9)  8 (89)  1 (11)  0 (0) | 0.9 (mg/dl) (0.7-1.3)  6 (60)  4 (40)  0 (0) | - 0.5 ^z^  FET | 0.63  0.30 |
| **Hematuria**  No  Microscopic occasional  Microscopic persistent  Macroscopic | 24 (100)  0 (0)  0 (0)  0 (0) | 16 (94)  1 (6)  0 (0)  0 (0) | FET | 0.41 |
| **Proteinuria**  Subnephrotic  Nephrotic | 7 (29)  17 (71) | 2 (12)  15 (88) | FET | 0.26 |
| **24-hour urinary protein** (gm/dl) | 4 (2 – 7.25) | 3.3 (2.3-5.5) | - 0.3 ^z^ | 0.75 |
| **S. Albumin** (gm/dl) | 2.9 (2.5-3.5) | 2.3 (1.9-2.8) | - 2 ^z^ | **0.05** |

Quantitative variables are expressed as median (interquartile range) for non-normally distributed data. Qualitative variables are expressed as number (percentage). BP: blood pressure, HTN: hypertension, S. Cr: serum creatinine, FET: Fisher’s exact test, s. albumin: serum albumin, z: Z-value, χ: chi square value

**Table 5S: Correlation between light microscopy findings of detached podocytes and response to steroid treatment in adults**

| **Response to steroids** | **Podocyte detachment by LM** | | **χ^2^**  **or**  **^FET^** | **P-value** |
| --- | --- | --- | --- | --- |
|  | **no** | **Yes** |  |  |
| **Early steroid response** (4 groups)  Complete remission  Partial remission  Steroid-dependent  No remission | 4 (37)  3 (27)  1 (9)  3 (27) | 0 (0)  3 (30)  1 (10)  6 (60) | FET | 0.17 |
| **Early steroid response** (3 groups)  Complete remission  Partial remission + steroid-dependent  No remission | 4 (36)  4 (36)  3 (28) | 0 (22)  4 (40)  6 (60) | FET | 0.1 |
| **Early steroid response** (2 groups)  Sensitive  Resistant | 8 (73)  3 (27) | 4 (40)  6 (60) | FET | 0.19 |
| **Late steroid response** (6 groups)  Complete remission  Partial remission  No remission  Infrequent relapsers  Frequent relapsers  Steroid-dependent | 0 (0.0)  2 (18)  4 (37)  1 (9)  3 (27)  1 (9) | 0 (0.0)  2 (20)  7 (70)  0 (0.0)  0 (0)  1 (10) | FET | 0.36 |
| **Late steroid response** (3 groups)  Complete remission + infrequent relapsers  Partial remission +frequent relapsers+ St. depend.  No remission | 1 (9)  6 (55)  4 (36) | 0 (0)  3 (30)  7 (70) | FET | 0.27 |
| **Late steroid response** (2 groups)  Sensitive  Resistant | 7 (64)  4 (36) | 3 (30)  7 (70) | FET | 0.19 |

Qualitative variables are expressed as number (percentage), FET: Fisher’s exact test

**Table 6S: Correlation between light microscopy findings and response to cyclosporine in adults**

| **Response to cyclosporine** | **Podocyte**  **detachment by LM** | | **χ^2^**  **or**  **^FET^** | **P-value** |
| --- | --- | --- | --- | --- |
|  | **no** | **Yes** |  |  |
| **Initial CSA response** (3 groups)  Complete remission  Partial remission  No remission | 6 (77)  3 (33)  0 (00) | 5 (62)  2 (12)  1 (25) | FET | 1 |
| **Late CSA response** (6 groups)  Complete remission  Partial remission  No remission  Infrequent relapsers  Frequent relapsers  CSA dependent | 5 (56)  3 (33)  0 (0)  0 (0)  1 (11)  0 (0) | 4 (50)  2 (25)  1 (12.5)  1 (12.5)  0 (0)  0 (0) | FET | 1 |
| **Late CSA response** (2 groups)  Sensitive  Resistant | 9 (100)  0 (0) | 7 (88)  1 (12) | FET | 0.47 |
| **Late CSA response** (3 groups)  Complete remission + infrequent relapsers  Partial remission+ frequent relapsers+ CSA depend.  No remission | 5 (56)  4 (44)  0 (0) | 4 (50)  3 (37.5)  1 (12.5) | FET | 1 |

Qualitative variables are expressed as number (percentage). CSA: cyclosporine A, FET: Fisher’s exact test

**Table 7S: Correlation between light microscopy findings of detached podocytes and clinical data in children**

| **Clinical data** | **Podocyte detachment by LM** | | **χ^2^/FET or**  **Z-value** | **P-value** |
| --- | --- | --- | --- | --- |
|  | **No** | **Yes** |  |  |
| **BP at presentation**  Normal  High | 34 (100)  0 (0) | 23 (85)  4 (15) | FET | **0.03** |
| **S. Cr at onset** (mg/dl)  ≤ 1 (mg/dl)  1.1 - 1.5 (mg/dl)  > 1.5 (mg/dl) | 0.5 (mg/dl) (0.5-0.7)  32 (94)  1 (3)  1 (3) | 0.5 (mg/dl)  (0.4-0.6)  24 (89)  2 (7)  1 (4) | - 0.97 ^z^  FET | 0.34  0.78 |
| **S. Cr after follow up** (mg/dl)  ≤ 1 (mg/dl)  1.1 - 1.5 (mg/dl)  > 1.5 (mg/dl) | 0.6 (mg/dl) (0.5-0.9)  13 (86)  1 (7)  1 (7) | 0.65 (mg/dl)  (0.5-0.8)  11 (92)  1 (8)  0 (0) | - 0.07 ^z^  FET | 0.94  1 |
| **Hematuria**  No  Microscopic occasional  Microscopic persistent  Macroscopic | 28 (82)  5 (15)  0 (0)  1 (3) | 24 (88)  1 (4)  1 (4)  1 (4) | FET | 0.28 |
| **Proteinuria**  Subnephrotic  Nephrotic | 7 (21)  27 (79.7) | 1 (4)  26 (96) | FET | 0.06 |
| **24-hour urinary protein** (gm/dl) | 2.5 (1.7 – 3) | 2 (1.8-3) | - 1.69 ^z^ | 0.09 |
| **S. Albumin** (gm/dl) | 2 (1.8-2.4) | 2 (1.5-2.3) | - 1.03 ^z^ | 0.3 |

Quantitative variables are expressed as median (interquartile range) for non-normally distributed data. Qualitative variables are expressed as number (percentage). BP: blood pressure, HTN: hypertension, S. Cr: serum creatinine, FET: Fisher’s exact test, s. albumin: serum albumin, z: Z-value.

**Table 8S: Correlation between light microscopy findings of detached podocytes and response to steroid treatment in children**

| **Response to steroids** | **Podocyte detachment by LM** | | **χ^2^**  **or**  **^FET^** | | **P-value** | |
| --- | --- | --- | --- | --- | --- | --- |
|  | **No** | **Yes** | |  | |  |
| **Early steroid response** (4 groups)  Complete remission  Partial remission  Steroid-dependent  No remission | 10 (34)  0 (0)  4 (13)  16 (53) | 5 (22)  0 (0)  4 (17)  14 (61) | | FET | | 0.69 |
| **Early steroid response** (3 groups)  Complete remission  Partial remission + steroid-dependent  No remission | 10 (34)  4 (13)  16 (53) | 5 (22)  4 (17)  14 (61) | | FET | | 0.69 |
| **Early steroid response** (2 groups)  Sensitive  Resistant | 14 (47)  16 (53) | 9 (39)  14 (61) | | 0.3 | | 0.58 |
| **Late steroid response** (6 groups)  Complete remission  Partial remission  No remission  Infrequent relapsers  Frequent relapsers  Steroid-dependent | 1 (3)  0 (70)  21 (70)  0 (0.0)  3 (10)  5 (17) | 0 (0.0)  0 (0.0)  19 (83)  0 (0.0)  1 (4)  3 (13) | | F.E.T | | 0.78 |
| **Late steroid response** (3 groups)  Complete remission + infrequent relapsers  Partial remission +frequent relapsers+ St. Depend.  No remission | 1 (3)  8 (27)  21 (70) | 0 (0)  4 (17)  19 (83) | | FET | | 0.61 |
| **Late steroid response** (2 groups)  Sensitive  Resistant | 9 (30)  21 (70) | 4 (17)  19 (83) | | 1.1 | | 0.29 |

Qualitative variables are expressed as number (percentage), FET: Fisher’s exact test. Seeking a significant correlation, we tried to re-group the patients according to steroid and CSA response in different ways. Analysis was carried out first for the small groups of partial responders, dependent, infrequent and frequent relapsers each in a separate group. Then we combined them into two large groups; a) **Sensitive**: including complete, partial remission, steroid/CSA dependent, frequent relapsers, and infrequent relapsers. B) **Resistant:** including cases with no remission.

**Table 9S: Correlation between light microscopy findings and response to cyclosporine in children**

| **Response to cyclosporine** | **Podocyte detachment by LM** | | | **χ^2^**  **or**  **^FET^** | | **P-value** |
| --- | --- | --- | --- | --- | --- | --- |
|  | **No** | **Yes** |  | |  | |
| **Initial CSA response** (3 groups)  Complete remission  Partial remission  No remission | 6 (50)  6 (50)  0 (0) | 7 (50)  7 (50)  0 (0) | FET | | 1 | |
| **Late CSA response** (6 groups)  Complete remission  Partial remission  No remission  Infrequent relapsers  Frequent relapsers  CSA dependent | 5 (42)  5 (42)  1 (8)  0 (0)  1 (8)  0 (0) | 5 (36)  8 (57)  0 (0)  1 (7)  0 (0)  0 (0) | FET | | 0.56 | |
| **Late CSA response** (2 groups)  Sensitive  Resistant | 11 (92)  1 (8) | 14 (100)  0 (0) | FET | | 0.46 | |
| **Late CSA response** (3 groups)  Complete remission + infrequent relapsers  Partial remission+ frequent relapsers+ CSA depend.  No remission | 5 (42)  6 (50)  1 (8) | 6 (43)  8 (57)  0 (0) | FET | | 0.83 | |

Qualitative variables are expressed as number (percentage). CSA: cyclosporine A, FET: Fisher’s exact test. Seeking a significant correlation, we tried to re-group the patients according to steroid and CSA response in different ways. Analysis was carried out firstfor the small groups of partial responders, dependent, infrequent and frequent relapsers each in a separate group. Then we combined them into two large groups; a) **Sensitive**: including complete, partial remission, steroid/CSA dependent, frequent relapsers, and infrequent relapsers. B) **Resistant:** including cases with no remission.

**Table 10s:** **Multivariate logistic regression analysis for prediction of late steroid resistance**

| **Variable** | **B** | **S. E.** | **Wald** | **P-value** | **OR** | **95 C.I. for OR** | |
| --- | --- | --- | --- | --- | --- | --- | --- |
|  |  |  |  |  |  | **Lower** | **Upper** |
| *Age:*  Children  Adult | -0.851 | 0.693 | 1.508 | 0.219 | R  0.427 | R  0.110 | R  1.661 |
| *Sex:*  Female  Male | -0.041 | 0.650 | 0.004 | 0.950 | R  0.960 | R  0.268 | R  3.434 |
| S. Alb (gm/dl) | -0.656 | 0.530 | 1.530 | 0.216 | 0.519 | 0.184 | 1.467 |
| LM podocyte detachment:  No  Yes | 1.495 | 0.683 | 4.798 | **0.028** | R  4.461 | R  1.170 | R  17.006 |

R: reference, OR: odds ratio, S. E: standard error, S. Alb: serum albumin, C.I.: confidence interval
